# The health-related quality of life in patients with prostate cancer managed with active surveillance using the Expanded Prostate Cancer Index Composite survey: Systematic review and meta-analysis

**DOI:** 10.1080/2090598X.2021.2024368

**Published:** 2022-02-27

**Authors:** Ahmed Abdelhafez, Khaled Hosny, Ahmed R. El-Nahas, Matthew Liew

**Affiliations:** a Wrightington Wigan and Leigh Teaching Hospitals NHS Foundation Trust, Wigan, UK; b James Cook University Hospital, Middlesbrough, UK; c Urology and Nephrology Centre, Mansoura University, Mansoura, Egypt

**Keywords:** Prostate cancer, health-related quality of life, Expanded Prostate Cancer Index Composite

## Abstract

**Objective:**

To examine the health-related quality of life (HRQoL) of patients with prostate cancer managed with active surveillance (AS) compared with those who receive definitive treatment using the Expanded Prostate Cancer Index Composite (EPIC) Survey.

**Methods:**

We used the Preferred Reporting Items for Systematic Reviews and Meta-Analyses (PRISMA) guidelines and searched PubMed and ScienceDirect for articles published between April 2010 and April 2020. Eligible studies reported original data on the HRQoL of men undergoing AS for prostate cancer, including studies comparing AS to curative methods particularly radical prostatectomy, radiotherapy, and brachytherapy.

**Results:**

We identified nine eligible articles, all were non-experimental observational studies of which seven were longitudinal and two were cross-sectional studies. The EPIC questionnaire was the main instrument used in all studies to assess the HRQoL. AS was noted to show the highest calculated mean score among management groups in all comparative studies at study endpoints including cross-sectional studies (95% confidence interval 2.17–5.75, *P* < 0.001). The maximum score deterioration for patients who were managed with AS in all studies was only 7.5 points (12.2%) after 2 years follow-up. AS had the least mean score decline among all management groups. Patients with a normal testosterone level were found to have high HRQoL scores. The number of prostate biopsies did not correlate with the HRQoL score.

**Conclusion:**

Patients with prostate cancer managed with AS report less impacts on their HRQoL compared to patients who receive definitive treatments. However, further high-quality research with long-term data are required to help both the patient and the physician in making a well-informed management decision.

## Introduction

Prostate cancer is the most common cancer among men in the UK in 2017 accounting for 26% of new cancer cases in males. Prostate cancer was the second most common cause of cancer mortality in the same year. Between 2013 and 2017, the estimated survival rate of men diagnosed with prostate cancer was 77.6% in England. PSA testing helped to improve prostate cancer survival in the UK by three folds in the last 40 years [1,2].

Radical prostatectomy (RP), external beam radiotherapy (EBRT), and brachytherapy (BT) are the mainstay treatment of localised prostate cancer. However, these modalities have detrimental effects on the patient’s quality of life [3,4]. Active surveillance (AS) was developed to allow patients with indolent prostate cancer to avoid the side-effects of definitive treatment, without losing the chance of having active management when indicated [4]. In AS patients, prostate biopsies are routinely used as a confirmatory tool; however, multiparametric MRI (mpMRI) of the prostate has been addressed by recent National Institute for Health and Care Excellence (NICE) guidelines as an alternative test [5,6].

Men eligible for AS have a low tumour volume, low malignant potential, and low PSA level at the time of diagnosis. Eligibility criteria from different guidelines and institutions can be found in the Table 1.

**Table 1.** Eligibility criteria for AS according to different guidelines and institutions

Guideline/Institution	Grade	Clinical stage	Serum PSA level, ng/mL	Biopsy Gleason	PSA density	Positive cores	Maximum cancer extent per core
NICE	Low Intermediate	T1–T2a T2b	˂10 10–20	≤6 7	NA	NA	NA
Royal Marsden		T1–2	≤15	≤3 + 4	–	˂50% of total cores	-
European Association of Urology (EAU)		T1c–T2	≤10	≤6	NA	≤2	≤50%
National Comprehensive Cancer Network (NCCN_	Very low Low	T1c T1–T2a	≤10 ≤10	≤6 ≤6	≤0.15 NA	˂50% NA	NA
AUA	Low Intermediate High	T1–T2a T2b T2c	≥10 ˃10–20 ˃20	≤6 7 8–10	NA	NA	NA
Memorial Sloan-Kettering Cancer Center (MSKCC)		T1–T2a	≤10	≤6	–	≤3	≤50%

Health-related quality of life (HRQoL) in prostate cancer aims to measure the physical and psychological elements required to assess patient status including urinary function, sexual function, bowel function, hormonal function, and associated bother [7–9].

The key principle of AS is to minimise the potential deterioration of the physical HRQoL in patients with prostate cancer compared to others who received definitive treatment. However, anxiety resulting from delayed therapy that affects the mental health of AS patients’ needs to be reduced as well to provide balanced management. Therefore, lots of studies that examine short- and long-term HRQoL have been conducted to help patients weigh the benefits and hazards of AS compared to other treatment strategies [10].

The Prostate Testing for Cancer and Treatment (ProtecT) trial was the first randomised clinical trial to compare treatment methods for localised prostate cancer in the PSA era [11]. It represents Level 1 evidence on the disease-specific HRQoL of the concurrent management options of prostate cancer. The study reported that AS had the least impact on disease-specific HRQoL of patients with prostate cancer at 6 years of follow-up. RP caused the highest negative impacts on urinary continence and sexual function, while EBRT was associated with more bowel dysfunction [3].

Plenty of HRQoL instruments have been developed to address the components of prostate cancer QoL based on the domains of urinary, bowel, and sexual function. The Expanded Prostate Cancer Index Composite (EPIC) survey is a QoL instrument that was developed from the original University of California-Los Angeles Prostate Cancer Index (UCLA-PCI) by a panel of experts of Urology oncologists, Radiation oncologists, survey researchers and prostate cancer nurses [3]. The original UCLA-PCI was enhanced by adding more items to assess extra urinary and bowel symptoms, haematuria, and hormonal symptoms. Symptom-specific bother items were added to the questionnaire in order to address a bother scale. The total score is calculated based on scores derived from each item from 0–100, with higher scores representing better QoL [12].

The other validated disease-specific instruments developed for HRQoL in patients with prostate cancer, e.g. the Functional Assessment of Cancer Therapy-Prostate (FACT-P) and European Organisation for Research and Treatment of Cancer quality of life questionnaire prostate specific 25-item (EORTC QLQ PR-25), are limited in evaluating obstructive and irritative voiding symptoms. They might be also lacking the assessment of both functions related bother and scores evaluating the effects of hormonal therapy. The EPIC is thought to offer a satisfactory survey instrument and an adequate test–retest reliability for urinary, bowel, sexual, and hormonal domain scores [13–21].

The aim of the present work was to provide a systematic review and meta-analysis of the studies that employed the EPIC survey to evaluate the impact of AS on the HRQoL of patients with prostate cancer. The EPIC instrument was chosen particularly in this review (as the main tool to assess the physical element) as it is comprehensive and widely used in the literature, therefore it would allow a meaningful comparison between different studies.

## Methods

### Literature search

The electronic databases of PubMed and ScienceDirect were selected to examine the literature. Systematic searches were done according to the Preferred Reporting Items for Systematic Reviews (PRISMA) standards. Search of the databases used the keywords ‘prostate cancer’, ‘cancer’, ‘active surveillance’, ‘health-related quality of life’, and ‘quality of life’. The keywords were typed in each database in different combinations that were chosen by the reviewers. The reviewers followed the same pattern in searching the databases to ensure the consistency of the data search. A search of the databases took place during April 2020 to capture relevant articles for the review.

### Eligibility criteria

The inclusion criteria of this review were articles published between April 2010 and March 2020, written in English language, and focussed only on the HRQoL of men undergoing AS. Types of studies included were randomised and non-randomised, comparative, and non-comparative, where data were collected either prospectively or retrospectively following the start of primary intervention for prostate cancer or patient enrolment in an AS protocol. They should have a sample size of ≥20 patients in each arm, reported HRQoL outcome measured using the EPIC survey with a minimal 12-month follow-up.

The population included adult men diagnosed with clinically localised prostate cancer, who had been managed with either AS or active treatment including EBRT, BT, or RP. Patients without cancer were involved in some studies as a reference. The exclusion criteria included commentaries, dissertations, theses, editorials, letters to the editor, books, and review articles.

**Figure 1.**
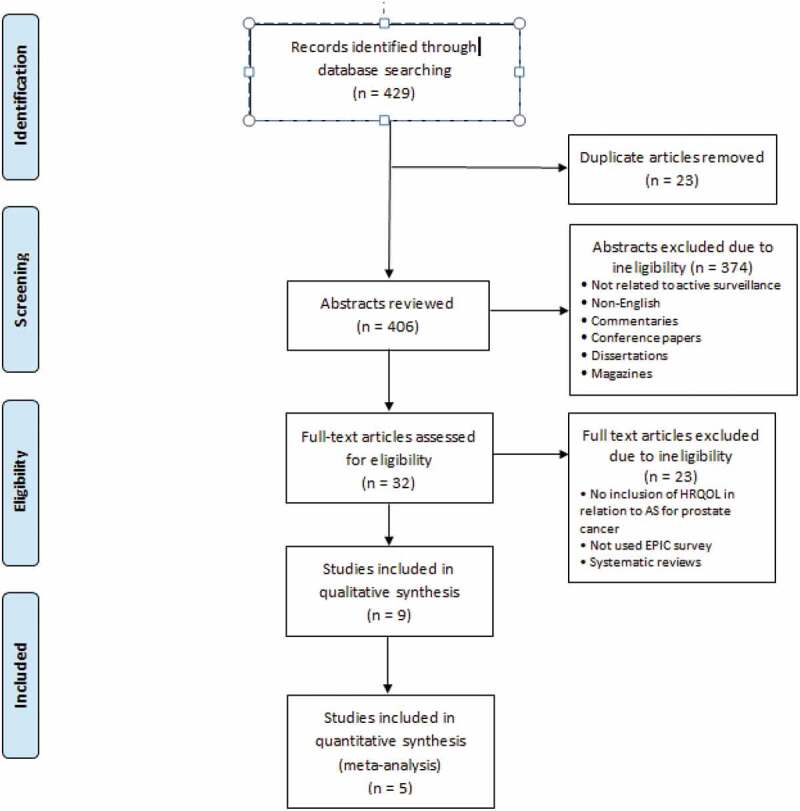
Data flow chart of article selection process.

The results of the search were used to identify the articles that met the inclusion criteria then further analysis was conducted to meet the objectives of the review. RevMan software version 5.4 [22] was used to for a pooled analysis for five studies comparing AS to definitive treatment. Figure 1 represents a flow chart of the process followed in the article’s selection.

The primary outcome of the present review was specified as the HRQoL score in patients with low-risk prostate cancer who were managed either by AS or active treatment measured using the EPIC survey. Secondary outcomes were the domains of prostate cancer-specific HRQoL such as urinary, sexual, and bowel function.

## Results

As shown in Figure 1, the search of the selected electronic databases yielded 429 articles. After exclusion of all ineligible articles, a thorough review of the remaining articles yielded a final nine articles that met all the inclusion criteria for the present review. Five studies were included in the pooled analysis. Risks of bias are shown in Figure 7.

All studies were non-experimental observational studies with seven longitudinal and two cross-sectional studies. Six articles included comparative groups and the other three articles focussed only on AS patients. Two studies involved non-cancer groups as references. The sample size in the studies ranged from 163 to 879. The racial distribution of the studies mostly consisted of White men and revealed a significant lack of minority populations. For example, the racial distribution for the study conducted by Parker et al. [23] was 86.1% White, 6.7% Black, 6.1% Hispanic, and 1.1% Asian, while the race was not reported in three articles.

The main questionnaire utilised in those studies was the EPIC, either the complete version (EPIC-50) or the short version (EPIC-26); however, other additional questionnaires were used such as Short-Form 36 Health Survey Questionnaire (SF-36) and SF-12 in seven studies. Other questionnaires were used such as the State-Trait Anxiety Inventory (STAI) and Mishel Uncertainty Illness Scale (MUIS). Five of the nine articles focussed on the combination of the HRQoL factors of sexual, bladder, and bowel function, and anxiety and depression. Two of the remaining four articles focussed on sexual dysfunction and testosterone levels, and the other two articles discussed HRQoL related anxiety and psychological well-being.

### *Comparative studies* (Figures 3 and 4)

#### AS vs RP vs EBRT vs BT

A cross sectional study by Sureda et al. [24] randomly matched two different groups of patients according to risk group, age, and time from start of treatment until completion of the survey. A group of 99 patients on AS for low-/intermediate-risk prostate cancer diagnosed between January 2008 and June 2015 was matched to an equal group from the Spanish Multicentric Study of Clinically Localised prostate cancer who underwent active treatment between 2003 and 2005.

The AS group reported lower physical and mental health score especially in bodily pain and vitality on short-term follow-up, which was explained by repeated prostate biopsies. The AS group reported better urine control scores compared to RP. The AS group at ≤2.5 years had better sexual function scores compared to all other groups but significantly better than RP. Other urinary and bowel function domains were equal in all groups.

The RP group reported better physical health dimensions than AS for <2.5 years but no difference at >2.5 years. While in the mental dimensions, the only difference was in the vitality score that was worse in the AS group who are aged ≤70 years or monitored for ≤2.5 years.

In another cross-sectional study by Venderbos et al. [25], they used an age-matched reference group of men without prostate cancer. Participants in the study were recruited from the European Randomized Study of Screening for Prostate Cancer (ERSPC) trial and from clinical practice with low-risk prostate cancer. Questionnaires were mailed to the patients and all patients had a minimum follow-up of ≥4 years.

The AS and RT groups reported better urinary functions than the RP group. The AS and reference groups reported less urinary incontinence than the RT and RP groups. The RT patients reported less urinary incontinence than the RP patients. Urinary bother and bowel function were similar in the four groups. The reference group reported no statistically significant difference in comparison to AS group for urinary continence and sexual functioning. Sexual function was highest in the AS group and lowest in the RP group. The AS group reported better physical summary scores than the RT group but was not clinically significant. There was no difference in the mental score in all groups. Interestingly, the reported level of generic anxiety was similar in the four groups.

The third study by Egger et al. [26], aimed at assessing the long-term psychological impact of conservative treatment of prostate cancer on patient’s HRQoL. They underwent a 10-year follow-up of low-risk prostate cancer patients who were managed with AS/watchful waiting (WW) in comparison to other groups managed initially with either RP, RT/high-dose rate (HDR) BT or low-dose-rate (LDR) BT.

In this study, AS/WW patients had equivalent long-term urinary bother and sexual health scores to patients who received immediate active treatment but with less bowel bother than RT/HDR BT patients and better urinary incontinence than RP patients. Initially, AS/WW patients had higher levels of distress than other groups. However, in general, AS/WW patients have similar long-term psychological QoL as the active treatment group. In this study, it was noted that a high percentage of patients under AS/WW (43%) received treatment during follow-up.

#### AS vs EBRT

Benarji et al. [27] recruited 60 patients treated with EBRT and 103 patients managed with AS. This longitudinal study had a mean follow-up of 44 months for EBRT and 32 months for AS using the EPIC and SF-36 questionnaires administered at baseline, every 3 months in the first year, then every 6 months.

They reported significantly worse bowel function at year 1 and 2 in the EBRT group. Both groups had similar bowel function and bother scores at 3 years. The EBRT group had a significant decline in the physical health score in the first 2 years after treatment. While the sexual function score showed an insignificant decline at the 2- and 3-year follow-up. It was noted that AS patients had a remarkable decline in urinary function at year 3.

#### AS versus RP

Jeldres et al. [9] recruited 228 patients who underwent RP and 77 patients managed with AS. Patients were aged ≤75 years with low-risk prostate cancer. The EPIC and SF-36 questionnaires were used at baseline and at different intervals up to 3 years.

The RP group had lower urinary function scores up to 3 years, with worse urinary bother scores in the first 18 months, but equivalent to AS at 2 and 3 years. Bowel function and bother scores became equal to AS at 2 years. There was no difference in hormonal functions. The AS group had better sexual function than the RP group at all times despite some improvement in the RP group at 6 months and 2 years but remained lower than AS at 3 years.

The SF-36 questionnaire outcome reported lower physical component score for RP at 3 months but equivalent to AS at 1 and 2 years, with no significant difference at 6 months. However, mental scores did not show any significant difference between the two groups.

#### AS negative prostate biopsy

Pham et al. [28] compared 89 patients diagnosed with low-risk prostate cancer and managed with AS to another group of 420 patients who had negative prostate biopsies. The EPIC and SF-36 surveys were conducted at baseline (before or after biopsy) then at 12, 24 and 36 months after biopsy.

Bowel functions were better in the AS group at 1 year but no significant difference afterwards. The AS group also showed a slight decline in urinary function and mental health, but it was insignificant and did not show meaningful clinical difference at 3 years.

### *Non-comparative studies* (Figures 5 and 6)

#### AS and sexual function

Pearce et al. [29] did a prospective study of 195 patients with low-risk prostate cancer on AS aiming at evaluating the relationship between repeat prostate biopsies and sexual dysfunction to predict the predisposing factors for sexual dysfunction. Patients did not receive any prior treatment for prostate cancer or prostate medications other than finasteride or dutasteride. Participants completed questionnaires at enrolment followed by a confirmatory biopsy, PSA and DRE and this was repeated every 6 months for 2 years with subsequent biopsies done every 2 years unless indicated by abnormal PSA or DRE.

They reported a decline in sexual score over the first 24 months. It was noted that length of time on AS, older age at enrolment, and diabetes, were independent risk factors for sexual dysfunction. While anxiety, body mass index (BMI), and number of biopsies did not predict sexual dysfunction. Interestingly, higher baseline PSA was associated with a more rapid decline in sexual function.

Cohen et al. [30] prospectively evaluated the impact of low testosterone levels on HRQoL of patients with prostate cancer on AS. They interviewed 223 patients with a mean age of 66.8 years who had low-risk/favourable-intermediate-risk prostate cancer. Questionnaires were answered at enrolment and every 6 months during the first 2 years of AS and annually thereafter. They defined low testosterone level as <300 ng/dL, low–normal 300–400 ng/dL, and normal ≥400 ng/dL according to the Endocrine Society Clinical Practice Guidelines that consider that a testosterone level of ˂300 ng/dL represents deficiency if accompanied by signs and symptoms of hypogonadism.

They reported that older age and obesity were associated with lower scores. Testosterone level is significantly correlated to patient satisfaction at 6 months, and increased testosterone level might be correlated to improved HRQoL in AS patients for at least 2 years. Patients’ stratification by testosterone level at enrolment helps to identify patients at risk of low HRQoL. In addition, they reported lower scores for urinary incontinence, urinary irritation, sexual and hormonal functions with lower testosterone levels; however, only incontinence was statistically significant.

#### AS and psychological function

Parker et al. [23] studied the impact of illness uncertainty, anxiety, fear of progression on HRQoL in patients with prostate cancer under AS. Different questionnaires were used in this study to evaluate the psychological well-being of the patients including STAI, MUIS, SF-12, and Memorial Anxiety Scale for Prostate Cancer. Patients were asked to complete questionnaires at the time of enrolment and every 6 months up to 30 months.

They reported that AS patients can maintain high HRQoL with minimal decline except for sexual function that decreased over time, but not clinically significant. Older age was associated with lower Physical Component Scale (PCS), sexual satisfaction scores, while higher BMI was associated with poorer hormonal, satisfaction, Physical Component Scale and Mental Component Scale scores. The number of prostate biopsies did not affect the sexual functioning score. Illness uncertainty decreased over time in AS starting from 12 months after study enrolment. Anxiety was higher at 12 and 24 months and lower at 18 and 30 months

### Quantitative assessment and main analysis

We conducted a limited meta-analysis only on the five comparative studies that compared AS to the definitive treatment options including RP, EBRT, and BT. The studies included 2599 participants with different comparisons of active treatments to AS. RevMan software version 5.4 [22] was used for the pooled analysis of the five studies. An index *I*^2^ between 0% and 40% was defined as not important heterogeneity, *I*^2^ between 30% and 60% was defined as moderate heterogeneity, *I*^2^ between 50% and 90% was defined as substantial heterogeneity, and *I*^2^ between 75% to 100% is defined as considerable heterogeneity. Heterogeneity was measured using the chi-squared statistics (*P* = 0.05).

The AS group was noted to show the highest calculated mean score among management groups in all comparative studies at study endpoints (95% CI 2.17–5.75; *P* < 0.001). There was no heterogeneity *I*^2^ = 0%, *P* = 0.89.

**Figure 2.**
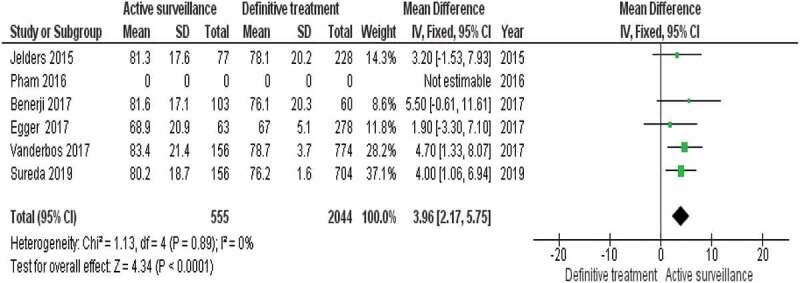
Pooled analysis table and Forest plot of the comparative studies.

A completed summary of the pooled analysis table and forest plot of the comparative studies can be found in Figure 2.

**Figure 3.**
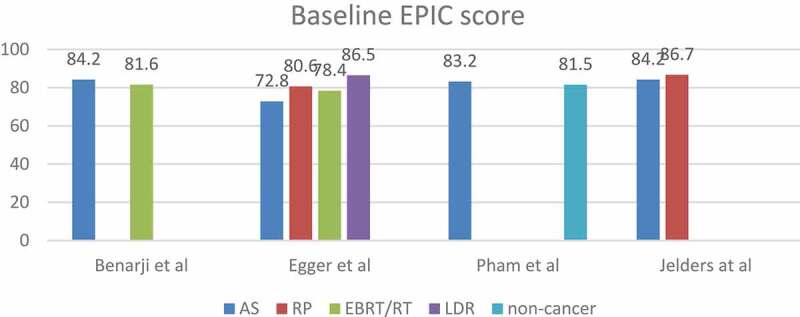
The mean HRQoL scores at baselines of the comparative studies.

**Figure 4.**
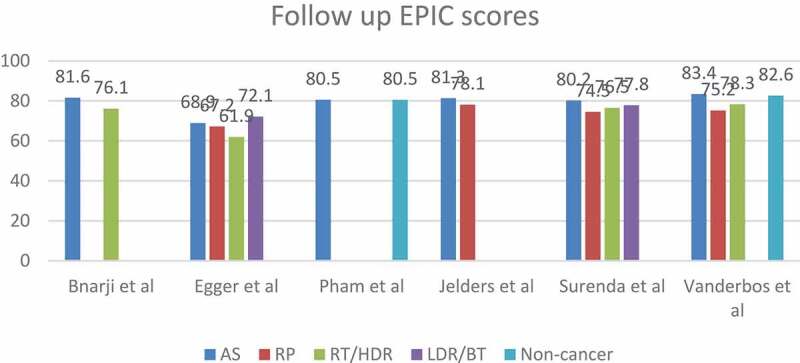
The mean HRQoL scores at the comparative studies end-points.

**Figure 5.**
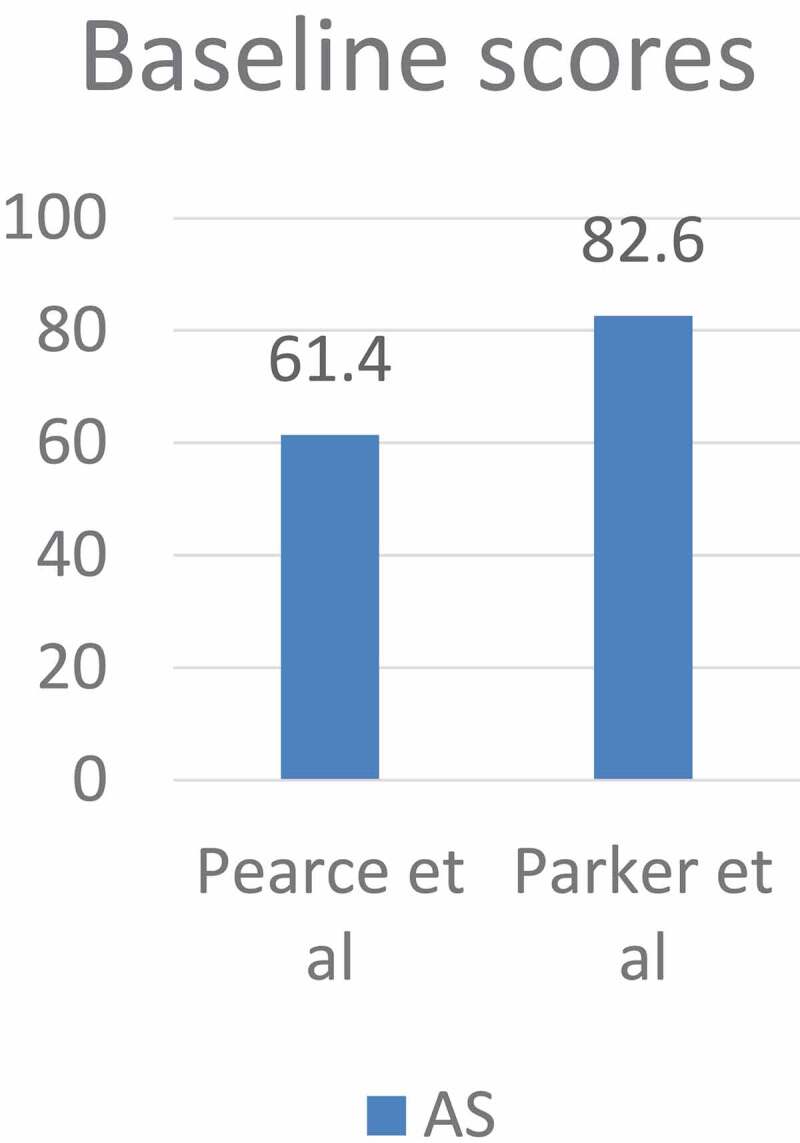
The mean HRQoL scores during AS at baselines of the non-comparative studies.

**Figure 6.**
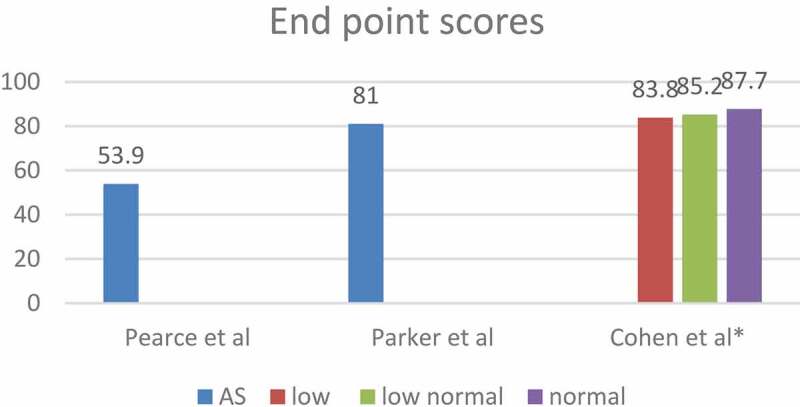
The mean HRQoL scores during AS at the non-comparative studies endpoints.

**Figure 7.**
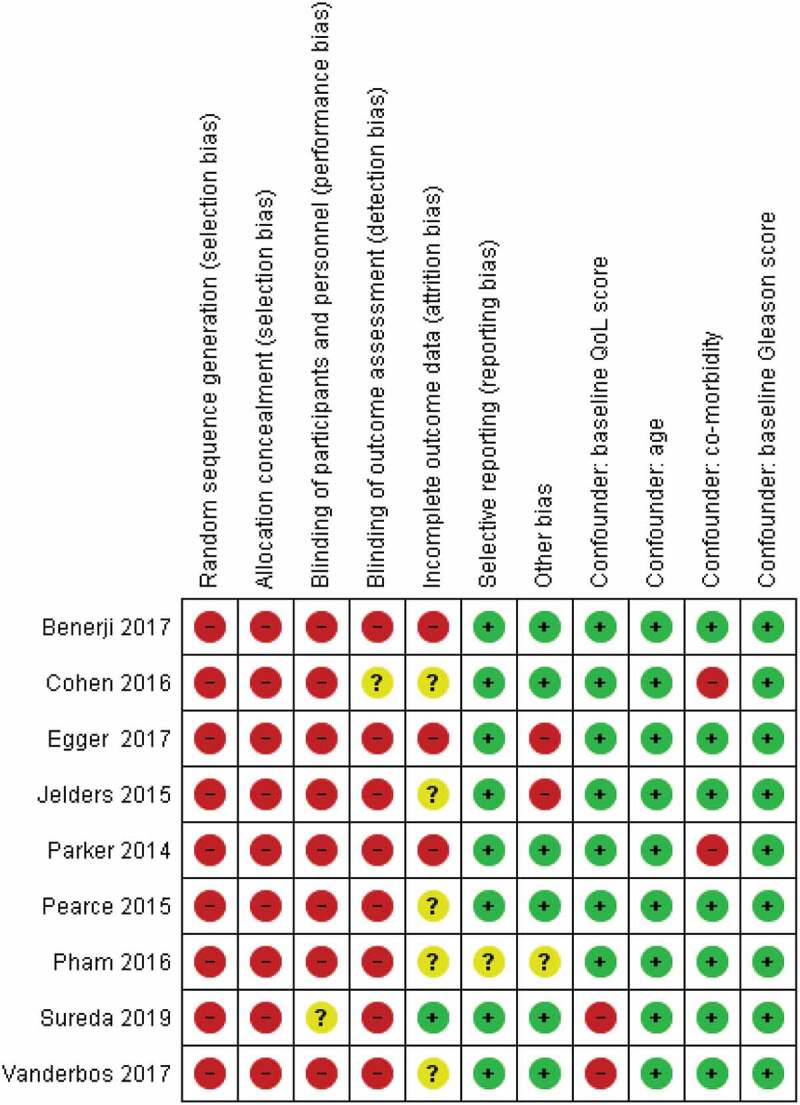
Risk of bias and confounding assessment. Red colour indicates high ROB, yellow uncertain ROB, and green low ROB. ROB, risk of bias.

#### Assessment of risk of bias and confounding factors

Two review authors independently assessed the risk of bias of each included study against key criteria: random sequence generation, allocation concealment, blinding of participants, personnel, and outcome; incomplete outcome data, selective outcome reporting and other sources of bias in accordance with methods recommended by The Cochrane Collaboration [8]. Additional items were added to assess the risk of confounders. Those confounders were developed by experts from the European Association of Urology Prostate Cancer Guideline Panel. The selected confounding factors consisted of age, baseline QoL score, baseline Gleason score, and comorbidities. The author’s judgements were categorised as either high risk, low risk, or unclear, which might be related to lack of information or uncertainty over the potential for bias. Disagreements between the reviewers were resolved by discussion and consensus.

## Discussion

Urinary function scores were generally worse in the surgically treated groups among all other groups in all studies and that was probably related to the highest prevalence of urinary incontinence. Urinary bother scores were not as low as urinary function. Urinary bother was even better in the surgical group than AS/WW in the Egger et al. [26] study, which is explained by older age in the WW group who are more bothered by other urinary symptoms. In patients under AS, urinary functions scores are equivalent to those of non-cancer groups and better than patients scores in the curative groups.

Patients undergoing treatment with RT had the highest prevalence of bowel dysfunction and as a result the worst scores in comparison to other treatment groups or non-cancer patients. The only exception was the LDR BT group in the Egger et al. [26] study who recorded the highest score, which suggests that LDR BT has the least long-term impact on bowel function.

Patients managed with AS had the best sexual function scores in comparison to patients receiving active treatment. However, the score dropped during follow-up in all studies even in the non-cancer group of Pham et al. [28]. This was accompanied with a similar drop in the sexual bother. Parker et al. [23] reported that older age has a negative influence on sexual function and satisfaction. Conversely, Korfage et al. [31] did not find a significant reduction in sexual bother among older-aged patients. This is explained by ‘response shift’, which means that those men underwent cognitive adaptation and accepted changes in their sexual functioning being an inevitable consequence of prostate cancer [31–33], which also explained the unexpected result of the Pham et al. [28] study.

The correlation between prostate biopsy and sexual dysfunction is controversial, as some studies reported no adverse effects of prostate biopsy on erectile function, while other studies indicate long- and short-term sexual dysfunction as a result of repeated prostate biopsies [34–36]. Pearce et al. [29] did not notice a correlation between the number of biopsies, the total Gleason score, and sexual dysfunction. However, their study was limited by the relatively small number of biopsies due to the short follow-up; as ~60% of patients had only one biopsy, therefore the impact of multiple biopsies could not be evaluated properly.

The most significant drop in the HRQoL score among all management groups was in the EBRT group. Egger et al. [26] reported that the mean score dropped by 16.5 points (21%) in the 10-year follow-up. Whilst Pearce et al. [29], reported that the maximum score deterioration for patients managed with AS was only 7.5 points (12.2%) after a 2-year follow-up. AS had the least mean score decline among all groups. Patients with normal testosterone levels are found to have better scores than others with low or low–normal levels.

AS showed the best mean total score in all comparative studies except in in the Egger et al. [26] study where LDR/BT had a better mean score. However, LDR/BT also showed a significant deterioration of the score with 14.4-point drop (16.6%) from the baseline score. The AS baseline score in the same study was lower than LDR/BT by 13.7 points and it dropped at the study end-point with only 3.9 points (5.3%). The LDR/BT high score at the study end-point might be explained by the small sample size (32 patients), therefore this should be interpreted cautiously.

In general, it can be observed that patients on AS perform well in most of HRQoL aspects, especially urinary and bowel functions. The physical and mental functions of AS patients can be affected by repeated prostate biopsies during follow-up; however, with the emergence of mpMRI in the new guidelines for monitoring as an alternative to repeat prostate biopsies, the impact will be reduced significantly. It does not seem that AS patients are suffering from more anxiety than other patients receiving curative treatment as would be expected. The level of anxiety and uncertainty seems to be easing off as time passes on AS. The impact on sexual function during AS is clearly caused by some factors that are unrelated to prostate cancer such as old age, diabetes mellitus, obesity, and testosterone level.

The available evidence has shown that the use of HRQoL questionnaires before and during AS is extremely useful in the clinical evaluation of the patients and understanding their needs and expectations. It would be good practice to discuss the impact of AS on the patient HRQoL in comparison to the curative treatment options during the counselling process before starting the patients on AS. The authors recommend the use of HRQoL questionnaires, such as the EPIC, before and after the start of AS.

Some limitations of the present study should be noted. First, only five of the nine studies included in this systematic review were comparative studies; therefore, we had to make a limited meta-analysis on the comparative studies. Another limitation is the relatively small number of cases in the included studies and the relatively short follow-up of patients, which did not exceed 36 months in most of the studies. Our future recommendation is to use other electronic databases not used in this systematic review to look at more results from different studies in literature.

## Conclusion

The evidence provided in this review shows that AS patients have the highest HRQoL EPIC scores in contrast to other patients treated with active treatment modalities. Patients with prostate cancer managed with AS reported less impacts on their HRQoL compared to patients who received definitive treatments.

Further high-quality research with long-term data is required to help both the patient and the physician in making a well-informed management decision.

## Abbreviations

AS: active surveillance

BT: brachytherapy

EORTC: European Organization for Research and Treatment of cancer

EPIC: Expanded Prostate Cancer Index Composite

FACT-P: Functional Assessment of Cancer Therapy-Prostate

HDR: high-dose rate

HRQoL: health-related quality of life

LDR: low-dose rate

MUIS: Mishel Uncertainty Illness Scale

NICE: National Institute for Health and Care Excellence

PRISMA: Preferred Reporting Items for Systematic Reviews

ProtecT: Prostate cancer for testing and treatment trial

RP: radical prostatectomy; (EB)RT: (external beam) radiotherapy

SF-12: Short-Form 12 Health Survey Questionnaire

SF-36: Short-Form 36 Health Survey Questionnaire

STAI: State-Trait Anxiety Inventory

UCLA-PCI: University of California-Los Angeles Prostate Cancer Index
